# Vaccine Mismatches, Viral Circulation, and Clinical Severity Patterns of Influenza B Victoria and Yamagata Infections in Brazil over the Decade 2010–2020: A Statistical and Phylogeny–Trait Analyses

**DOI:** 10.3390/v14071477

**Published:** 2022-07-05

**Authors:** Jaline Cabral da Costa, Marilda Mendonça Siqueira, David Brown, Jonathan Oliveira Lopes, Braulia Caetano da Costa, Eric Lopes Gama, Maria de Lourdes Aguiar-Oliveira

**Affiliations:** Laboratory of Respiratory Virus and Measles, Oswaldo Cruz Institute, Oswaldo Cruz Foundation. Av. Brasil, 4365 Manguinhos, Rio de Janeiro 21040-360, RJ, Brazil; mmsiq@ioc.fiocruz.br (M.M.S.); david.brown@phe.gov.uk (D.B.); jonathan.lopes@ioc.fiocruz.br (J.O.L.); braulia.caetano@ioc.fiocruz.br (B.C.d.C.); ericgama70@hotmail.com (E.L.G.)

**Keywords:** influenza B lineages, vaccine mismatch, clinical disease, phylogenetics, phylogeny–trait association

## Abstract

Worldwide, infections by influenza viruses are considered a major public health challenge. In this study, influenza B vaccine mismatches and clinical aspects of Victoria and Yamagata infections in Brazil were assessed. Clinical samples were collected from patients suspected of influenza infection. In addition, sociodemographic, clinical, and epidemiological information were collected by the epidemiological surveillance teams. Influenza B lineages were determined by real-time RT-PCR and/or Sanger sequencing. In addition, putative phylogeny–trait associations were assessed by using the BaTS program after phylogenetic reconstruction by a Bayesian Markov Chain Monte Carlo method (BEAST software package). Over 2010–2020, B/Victoria and B/Yamagata-like lineages co-circulated in almost all seasonal epidemics, with B/Victoria predominance in most years. Vaccine mismatches between circulating viruses and the trivalent vaccine strains occurred in five of the eleven seasons (45.5%). No significant differences were identified in clinical presentation or disease severity caused by both strains, but subjects infected by B/Victoria-like viruses were significantly younger than their B/Yamagata-like counterparts (16.7 vs. 31.4 years, *p* < 0.001). This study contributes to a better understanding of the circulation patterns and clinical outcomes of B/Victoria- and B/Yamagata-like lineages in Brazil and advocate for the inclusion of a quadrivalent vaccine in the scope of the Brazilian National Immunization Program.

## 1. Introduction

Globally, influenza infections are a major public health challenge due to morbidity and mortality and have a significant annual economic impact [1,2]. Influenza types A or B are clinically indistinguishable [3,4] and can lead to serious complications and death, especially among children and adults [5,6,7]. In Brazil, the burden of influenza-like illness (ILI) cases was estimated to be over 83 million in 2008 [8]. 

Annual vaccination plays a key role in influenza control and prevention [9]. Although several countries have regularly used the quadrivalent influenza vaccine (QIV) for some years now, the Brazilian Immunization Program freely provides the trivalent influenza vaccine (TIV), which comprises two strains of influenza A (H1N1 and H3N2) and only one influenza B lineage component-B/Yamagata or B/Victoria-like [10]. Because of the high viral variability, especially in hemagglutinin and neuraminidase proteins, the vaccine composition is annually updated by the World Health Organization (WHO), based on a sample of circulating viruses that are characterized by the global influenza surveillance network [11,12,13,14]. For the TIV, the B/Victoria or B/Yamagata-like strains are chosen according to their prevalence in each Hemisphere based on the prior year [14]. Therefore, the success of the vaccination strategy depends on the concordance between the recommended vaccine strain and their effective prevalence in the population in a given influenza season.

Since 2000, the co-circulation of influenza B lineages has been observed, imposing a challenge for TIV adoption [15]. In many countries, mismatches between vaccine and circulating viruses had been reported in about two-to four year intervals [16]. These events can contribute to additional disease-related burden due to the limited cross-protection between antigens [17,18,19]. Hence, the correct prediction of vaccine strains is pivotal to a successful immunization policy and the reduction in the annual impact of influenza. 

In this study, we investigated the distribution and presumed regional patterns of influenza B lineages among 11 influenza seasons (2010–2020) in Brazil. In addition, putative associations between B/Victoria- and B/Yamagata-like viruses and demographic and clinical-epidemiological variables were also explored. This information is critical to tailor public health policies for influenza control and prevention.

## 2. Materials and Methods

### 2.1. Population

Nasopharyngeal swabs, aspirates, and/or lung tissue fragments of 920 influenza B laboratory-confirmed cases were investigated. From June 2010 to March 2020, clinical samples were collected from subjects with respiratory influenza-like illness (ILI) or Severe Acute Respiratory Infection (SARI), according to the WHO and Brazilian Ministry of Health (MoH) case definitions [20,21] and sent to our laboratory, a reference laboratory for the MoH and WHO. In addition, the sociodemographic, clinical, and epidemiological information were collected by the local epidemiological surveillance teams using a nationally standardized questionnaire including information on gender, age, symptoms onset, clinical signs and symptoms, hospitalization history and comorbidities, and others.

Among the 920 influenza B positive samples used in the mismatch analysis, 514 samples had complete clinical and epidemiological data and were used to explore putative associations between influenza B lineages and those outcomes. In addition, within the subsample submitted to viral HA sequencing, good quality and complete sequences were obtained in 118 samples.

Clinical severity was defined as the presence of dyspnea, indicative of SARI. Samples were collected in different Brazilian geographical regions. The Northeastern states were represented by Alagoas, Bahia and Sergipe, whereas Southeastern and Southern states by Rio de Janeiro, Espírito Santo, and Minas Gerais, and Rio Grande do Sul, Paraná, and Santa Catarina, respectively. Vaccine mismatch was defined as more than 51% of divergence between the circulating lineage and influenza B vaccine strain in each influenza season.

### 2.2. Influenza B Molecular Detection, Lineage Determination and Sequencing

Viral RNA was extracted using the QIAmp Viral RNA Mini Kit. Lung tissue fragments were macerated using the Tissue Ruptor Kit and RNA was extracted using a RNeasy Mini Kit (Qiagen, Hilden, Germany). Influenza B detection and lineage determination were performed by real-time RT-PCR using the CDC protocols, as recommended by the WHO [22]. Sanger sequencing of the hemagglutinin gene (HA, 1714bp) was carried out using the CDC primers and protocol. After purification (QIAquick Extraction Kit, Qiagen), both strands were sequenced using the ABI PRISM BigDye Terminator v.3.1 Cycle Sequencing Ready Reaction Kit (Applied Biosystems, Waltham, MA, USA). 

### 2.3. Phylogenetic Analyses

The phylogenetic analyses were composed of 118 complete Brazilian HA sequences (1714bp), for which information on the presence of dyspnea was available. Sequences were edited and contigs were set up using the software Sequencher, v.4.10. After alignment by Muscle [23], HA phylogenetic trees were reconstructed using a maximum likelihood algorithm (PhyML v.3.0) and aLRT SH-like as the fast likelihood-based method [24]. The general time reversible with gamma-distributed rates and invariant sites (GTR + I+G) was employed as the best fit nucleotide substitution model determined by the J Model test, version 2.1.7 [25].

The temporal structure of the dataset was verified using TempEst v. 1.5.1 [26]. Afterward, time–scale phylogenetic trees were reconstructed by a Bayesian Markov Chain Monte Carlo (MCMC) method, accessible in the BEAST software package, v1.10 [27,28]. Time calibration was set based on the year of sample collection, available for all sequences. Beast runs were carried out using the uncorrelated lognormal relaxed molecular clock model and a time-aware Gaussian Markov Random Field (GMRF) Bayesian skyride coalescent tree prior [29,30]. The length of the MCMC chains was established as 80 million, sampled every 8000 steps. Trace files generated through Bayesian phylogenetic inference were visualized and analyzed in Tracer version 1.7.1 [31]. The convergence of parameters was considered in the presence of effective sample size (ESS) values exceeding 200. The target maximum clade credibility (MCC) tree was summarized by TreeAnnotator 1.8.4, with a burn-in corresponding to 10% of states. PhyML and MCC trees were visualized and edited in FigTree, version 1.4.3. (http://tree.bio.ed.ac.uk/software/figtree/ accessed on 31 July 2021).

In order to infer putative phylogeny–trait associations (viral lineages and disease severity), we used 100 replicates for two discrete states (ILI and SARI). Analyses were performed with BaTS program, release 0.9 [32]. Parsimony score statistics (PS), association index (AI), and monophyletic clade (MC) statistics were calculated. 

### 2.4. Statistical Analyses

Descriptive and bivariate analysis (chi-square/Fisher’s exact test for categorical variables and the independent-samples Kruskal–Wallis test for means) were employed to assess the putative associations between the variables of interest and outcomes. Significance was considered when the *p* value < 0.05. Analyses were performed using SPSS for windows, version 19 (SPSS Inc., Chicago, IL, USA).

### 2.5. Ethical Statement

This study was approved by the Fiocruz-IOC Ethics Committee (68118417.6.0000.5248). As a National Reference Laboratory for Influenza for the MoH and as a National Influenza Center (NIC) for the WHO, our laboratory continuously receives samples from influenza cases for antigenic and genetic characterization as part of the WHO Influenza Surveillance Network. Clinical samples were collected in the scope of the National Influenza Epidemiological Surveillance Program/MoH, dispensing a formal patient consent. 

In accordance with our confidentiality policy, determined in the scope of Quality System (ISO 15189), personal information is confidential, and all analyses remain anonymous as the samples and formularies are coded.

## 3. Results

The demographic and clinical features of the studied population, according to influenza B viral lineage strata are shown in Table 1. Most of the subjects of the male gender (54.2%), with a median age of 20.5 years (0–99 years), and residents in Southern Brazil (70.2%). About a third of the sample reported any comorbidity (28.5%) and a low frequency of fatal outcomes was observed (3.4%). With concern to clinical symptoms, about 40.0% of cases reported dyspnea, in line with the SARI case classification. The majority of patients presented fever and cough (about 93.0%), sore throat (48.0%), myalgia (26.6%), coryza (21.4%), and arthralgia (4.3%). Individuals infected by B/Victoria-like viruses were significantly younger than their B/Yamagata-like counterparts (16.7 vs. 31.4 years, *p* < 0.001). No other significant divergence in the demographic or clinical variables could be noted, suggesting a similarity in clinical disease caused by B/Victoria- and B/Yamagata-like infections. These 514 samples were also subtyped and 51.6% of infections were associated with Victoria-like viruses (*n* = 265), while the remaining 48.4% had Yamagata-like infections (*n* = 249).

**Figure 1:**
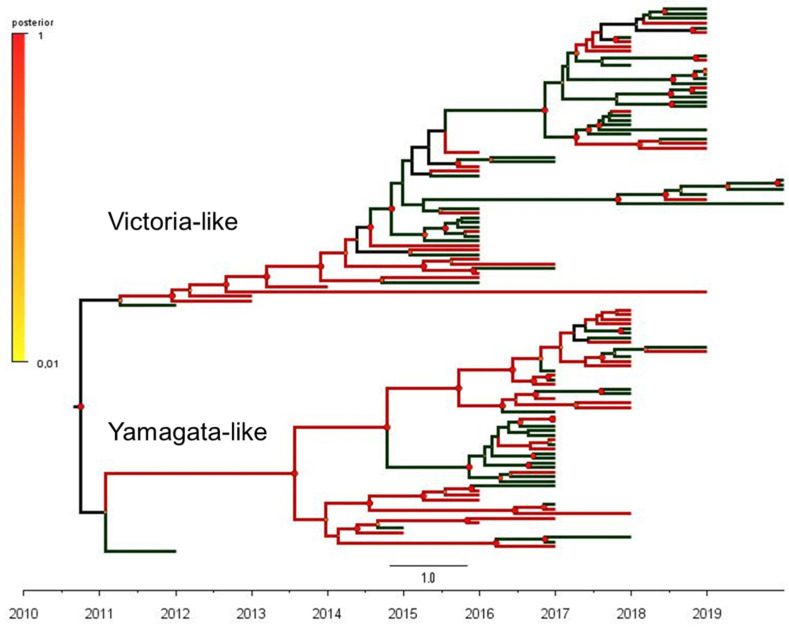
The phylogeny–trait analysis based on the maximum clade credibility (MCC) phylogenetic tree of 118 complete sequences of the influenza B hemagglutinin gene (1714 bp) from B/Victoria-like (66 sequences) and B/Yamagata-like (52 sequences) antigenic lineages circulating in Brazil, 2010–2020.

Full HA sequences and clinical information was available for a subsample of 118 positives (66 sequences of B/Victoria and 52 sequences of B/Yamagata-like), which were further classified as ILI or SARI-associated cases (Table S1). The maximum clade credibility (MCC) phylogenetic tree of the influenza B hemagglutinin gene (1714 bp) from B/Victoria- and B/Yamagata-like lineages circulating in Brazil and the outcomes of the phylogeny–trait analysis are shown in Figure 1. In order to effectively describe the putative correlations between traits (clinical severity, ILI or SARI) and phylogeny, we used a Bayesian tip-association significance test (Table 2). In these assessments, low PS scores and AI values represent strong phylogeny–trait association. In addition, MC values will be correlated with the strength of the phylogeny–trait association [32]. The comparison between the AI and PS values obtained in our subsample and the null mean values corresponded to 6.4 (95%CI 5.5–7.2) vs. 6.3 (95%CI 5.1–7.4), *p* = 0.550 and 37.8 (95% CI 36.0–40.0) vs. 38.9 (95% CI 34.9–43.1), *p* = 0.370), respectively. These outcomes revealed a weak phylogeny–trait association. In addition, the monophyletic clade statistics (MC) for SARI was 2.9 (95% CI 2.0–5.0) vs. 3.4 (95% CI 2.6–4.5), *p* = 0.810) and for ILI, it was 3.7 (95% CI 3.0–5.0) vs 4.4 (95% CI 3.1–6.2, *p* = 0.569), showing that the SARI and ILI traits were randomly distributed among the B/Victoria and B/Yamagata groups. Altogether, these results support the view that infections caused by B/Victoria-like and B/Yamagata-like viruses have similar clinical severity, independent of the hypothesis testing approach. 

**Figure 2:**
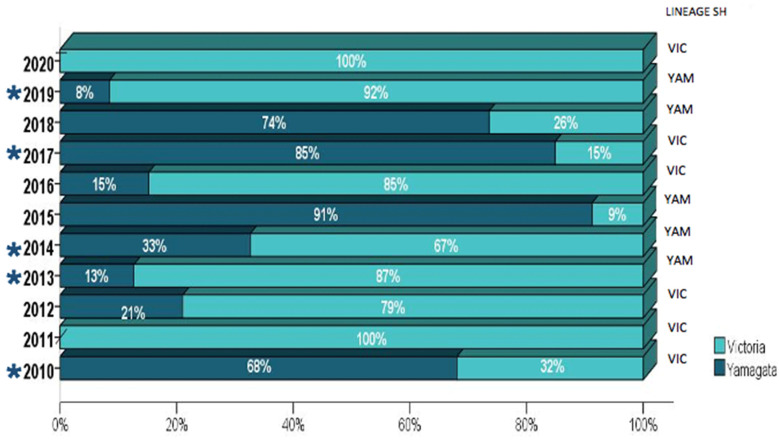
The distribution of influenza B lineages among the 920 Brazilian samples collected from 2010–2020. The number of tested samples for each year is further detailed in Table S2. The years where mismatches between the vaccine and circulating viruses were observed are indicated by (*).

The distribution of viral lineages according to the year of sample collection and the presence of vaccine mismatches is shown in Figure 2. Both influenza B lineages co-circulated through the studied decade, with a higher prevalence of B/Victoria-like viruses in most years. Mismatches between vaccine strains (Table 3) and circulating lineages (B/Victoria and B/Yamagata) were observed in 45.4% (5/11) of the investigated seasons (2010, 2013, 2014, 2017, and 2019). 

## 4. Discussion

In this study, the demographic and clinical aspects of influenza B/Victoria and B/Yamagata infections, and vaccine mismatches over a decade of influenza seasons were explored. 

Independent of the adopted hypothesis testing approach—if based on statistical analyses on the main influenza signs and symptoms or on phylogeny–trait analyses—our results revealed that infections caused by B/Victoria-like and B/Yamagata-like viruses presented similar clinical outcomes/severity. These findings are in line with previous reports [33,34,35,36]. Nonetheless, observations from Tan et al. suggest that the B/Victoria Guangzhou clade 2 lineage infected patients showed fewer upper respiratory tract infections than their B/Victoria Guangzhou clade 1 counterparts [37].

Of note, subjects infected by B/Victoria-like viruses were significantly younger than those infected by B/Yamagata-like viruses, corroborating data from epidemiological studies conducted in Brazil and elsewhere (Slovenia, Australia, New Zealand, and South Africa) [34,38,39,40,41]. In addition, previous analyses on demographic data (2008 to 2019) had shown a younger profile among B/Victoria (median age of 13 y) when compared to the B/Yamagata cases (median age of 32.5y)—the latter showing a bimodal age distribution with peaks within pediatric and adult age groups, respectively [42,43]. According to Vijaykrishna et al., this outcome could be partially explained by subtle differences in the prevalence of α-2,3 and α-2,6 linked glycans on respiratory tract cells from young children, in contrast to those found among adults [39], in addition to pre-exposure to infection or immunization combined with pre-existing population immunity. Moreover, the existence of an immunological impression induced by a first B/Yamagata infection could act on more conserved epitopes than those neutralized by antibodies induced by B/Victoria-like viruses [39,44,45]—a phenomenon already described for influenza A viruses [46,47].

In our analysis, no special geographical patterns in the distribution of viral lineages were found. Despite a profound imbalance in the demographic density between Brazilian regions [48], the reduced number of available sequences from the Northeastern states is noteworthy (Table 1), reinforcing the need to improve epidemiological and genomic surveillance, in order to have a representative sample of all regions. This is pivotal information to effectively evaluate the putative geographical patterns of viral distribution and to better guide vaccination strategies. It is important to emphasize that all of these findings should be interpreted in light of a small and non-representative sample, which could have introduced biases in the present analyses.

The presence of mismatches between the circulating influenza B lineages and vaccine strains in the 2010–2020 influenza seasons was also explored. The re-emergence of B/Victoria-like viruses and the cocirculation of both influenza B strains since 2000–2002 [49,50,51] including the sample assessed in this study imposed a challenge to a correct prediction of the TIV influenza B component. Our figures pointed to a vaccine mismatch in 45.5% of the studied seasons (2010, 2013, 2014, 2017, and 2019), in line with the previous Brazilian information [52,53,54,55]. Luna et al. described similar findings (46.0%) over seven influenza seasons (2010–2016) [38,55], and Barros et al. found a significant vaccine mismatch in 2013, both for Brazil (Vic 91.4%) and for South America (Vic 52%) [54]. A study carried out in Europe and in the United States showed vaccine mismatches in about half of the 2001 to 2011 influenza seasons [16]. It is relevant to mention that global sampling on the influenza surveillance program is not representative at all, and is based on the sample collection of sentinel health services [14,21]. Moreover, the effective impact of this event on influenza morbidity and mortality need to be further addressed.

Altogether, these results highlight the difficulty to accurately predict the influenza B component of the annual trivalent vaccines, despite the WHO global efforts to monitor and characterize circulating viruses in the Northern and Southern Hemispheres. In addition, influenza vaccine effectiveness may be suboptimal in mismatched seasons, potentially increasing the disease burden [56]. In order to reduce the impact of influenza B vaccine mismatch, the WHO has recommended the inclusion of both influenza B lineages in the vaccine composition since 2013 [57]. However, the TIV is under current use by the MoH Immunization Program to vaccinate influenza target groups [10]. The impact of replacing the TIV by QIV in a pediatric group has been estimated. When the dynamic epidemiological model was applied to the Brazilian context, QIV adoption would be able to avoid 406,600 symptomatic cases, 11,300 hospitalizations, and almost 400 deaths per influenza season, reinforcing the cost-effectiveness of QIV and its respective public health benefits [58,59,60,61].

## 5. Conclusions

Despite annual vaccination campaigns, seasonal influenza remains responsible for a relevant morbidity and mortality and economic burden in Brazil and worldwide. Overall, our findings advocate for the inclusion of the QIV in the context of the Brazilian National Immunization Program, in order to improve the health promotion and economic benefits of influenza vaccination.

## Figures and Tables

**Table 1:** Demographic and clinical features among the 514 Influenza B infected individuals, according to viral lineage in Brazil, 2010–2020.

		Influenza B Lineages		
**Variables**	**All (*n* = 514)**	**Victoria (*n* = 265)**	**Yamagata (*n* = 249)**	***p*-Value**
Brazilian geographical region (%)		51.6 (265)	48.4 (249)	0.157
Northeast	6.2 (32/514)	7.9 (21)	4.4 (11)	
Southeast	23.5 (121/514)	24.9 (66)	22.1 (55)	
South	67.2 (178/514)	73.5 (178)	73.5 (183)	
Gender (masc %)	54.2 (278/514)	57.4 (152)	50.8 (126)	0.081
Comorbidities (%)	28.5 (103/361)	29.9(49)	27.4 (54)	0.344
Hospitalization	90.4 (255/282)	92.6 (138)	88.0 (117)	0.131
Fatal outcome (%)	3.4 (14/409)	3.4 (6)	3.4 (8)	0.599
Reported clinical symptoms (%)				
Dyspnea	39.7 (204/514)	39.6 (105)	39.8 (99)	0.523
Fever	93.6 (480/513)	94.3 (249)	92.8 (231)	0.297
Cough	93.8 (480/512)	93.6 (247)	94.0 (233)	0.501
Sore throat	48.0 (243/506)	45.4 (118)	50.8 (125)	0.129
Myalgia	26.6 (136/511)	24.7 (65)	28.6 (71)	0.184
Coryza	21.4 (109/510)	24.1 (63)	18.5 (46)	0.073
Arthralgia	4.3 (22/512)	3.8 (10)	4.8 (12)	0.363
Age (median, range in years)	20.5 (0–99)	16.7 (0–99)	31.4 (0–88)	<0.001

**Table 2:** Results of phylogeny trait association tests for viral lineages (B/Victoria and B/Yamagata) and disease severity (SARI and ILI).

Statistic	Observed Mean (95%CI)	Null Mean (95%CI)	Significance (*p*-Value)
AI	6.4 (5.5–7.2)	6.3 (5.1–7.4)	0.550
PS	37.8 (36.0–40.0)	38.9 (34.9–43.1)	0.370
MC SARI	2.9 (2.0–5.0)	3.4 (2.6–4.5)	0.810
MC ILI	3.7 (3.0–5.0)	4.4(3.1–6.2)	0.569

AI, association index; PS, parsimony score; MC, monophyletic clade; CI, confidence interval; SARI, severe acute respiratory infection; ILI, influenza-like illness. The sequences obtained from SARI and ILI cases and represented in red and green, respectively. Results were regarded as statistically significant when *p* < 0.05.

**Table 3:** WHO recommended influenza vaccine composition in the respective influenza seasons.

Influenza Season	Northern Hemisphere (NH)		Influenza Season	Southern Hemisphere (SH)	
	Lineage	Strain		Lineage	Strain
2019/20	Yamagata	B/Phuket/3072/2013	2020	Victoria	B/Washington/02/2019
2018/19	Yamagata	B/Phuket/3072/20131	2019	Yamagata	B/Phuket/3072/20132
2017/18	Victoria	B/Brisbane/60/2008	2018	Yamagata	B/Phuket/3072/2013
2016/17	Victoria	B/Brisbane/60/2008	2017	Victoria	B/Brisbane/60/2008
2015/16	Yamagata	B/Phuket/3072/2013	2016	Victoria	B/Brisbane/60/2008
2014/15	Yamagata	B/Massachusetts/2/2012	2015	Yamagata	B/Phuket/3072/2013
2013/14	Yamagata	B/Massachusetts/2/2012	2014	Yamagata	B/Massachusetts/2/2012
2012/13	Yamagata	B/Wisconsin/1/2010	2013	Yamagata	B/Wisconsin/1/2010
2011/12	Victoria	B/Brisbane/60/2008	2012	Victoria	B/Brisbane/60/2008
2010/11	Victoria	B/Brisbane/60/2008	2011	Victoria	B/Brisbane/60/2008
2009/10	Victoria	B/Brisbane/60/2008	2010	Victoria	B/Brisbane/60/2008

## Data Availability

Not applicable.

## Supplementary Materials

The following supporting information can be downloaded at: https://www.mdpi.com/article/10.3390/v14071477/s1, Table S1: Sequences from GISAID’s EpiFlu™ Database, Table S2: Distribution of Influenza B lineages among 920 Brazilian samples collected from 2010–2020. The number of tested samples for each year and lineages are detailed.
